# Pediatric Diffuse Midline Gliomas H3 K27M-Mutant and Non-Histone Mutant Midline High-Grade Gliomas in Neurofibromatosis Type 1 in Comparison With Non-Syndromic Children: A Single-Center Pilot Study

**DOI:** 10.3389/fonc.2020.00795

**Published:** 2020-06-03

**Authors:** Federica Garibotto, Francesca Madia, Claudia Milanaccio, Antonio Verrico, Arnoldo Piccardo, Domenico Tortora, Gianluca Piatelli, Maria Cristina Diana, Valeria Capra, Maria Luisa Garrè, Andrea Rossi, Giovanni Morana

**Affiliations:** ^1^Neuro-oncology Unit, IRCCS Istituto G. Gaslini, Genova, Italy; ^2^Laboratory of Neurogenetics and Neuroscience, IRCCS Istituto G. Gaslini, Genova, Italy; ^3^Nuclear Medicine Unit, Ente Ospedaliero Ospedali Galliera, Genova, Italy; ^4^Neuroradiology Unit, IRCCS Istituto G. Gaslini, Genova, Italy; ^5^Neurosurgery Unit, IRCCS Istituto G. Gaslini, Genova, Italy; ^6^Pediatric Neurology and Muscular Diseases Unit, IRCCS Istituto G. Gaslini, Genova, Italy

**Keywords:** H3K27M, pediatric, brain tumor, diffuse midline glioma, NF1

## Abstract

**Background:** Pediatric neurofibromatosis type 1 (NF1) patients rarely develop aggressive central nervous system tumors. Among high-grade gliomas (HGGs), histone mutant diffuse midline gliomas (DMGs H3 K27M-mutant) have exceptionally been reported. The aim of this retrospectives single-center study was to compare the clinical behavior of DMGs H3 K27M-mutant and non-histone mutant midline HGGs in NF1 vs. non-syndromic children and to report imaging features of NF1 HGGs.

**Method:** We conducted a retrospective review of cerebral DMGs H3 K27M-mutant or non-histone mutant HGGs in 18 patients with or without NF1 followed at our institution between 2010 and 2018. Differences in outcomes, notably progression-free survival (PFS) and overall survival (OS), were evaluated.

**Results:** Two patients were identified with genetically confirmed diagnosis of NF1 and cerebral HGGs (one DMG H3 K27M-mutant and one histone wild type). Both subjects presented with midline mass lesions with imaging features of aggressive biological activity on advanced MRI or amino-acid PET. During the same time period, 16 non-NF1 patients (11 subjects with DMGs H3 K27M-mutant and 5 with non-histone mutant midline HGGs) were treated at our institution. The two patients with NF1 and HGGs presented a PFS of 3 months and an OS of 5 and 7 months. Median PFS and OS of children without NF1 were respectively 6 and 10 months in DMGs H3 K27M-mutant, and 6 and 11 months in H3 K27M wild-type tumors. Seventy-five percent of subjects with non-NF1 HGGs presented a PFS >4 months compared to 0% in NF1 patients. The 8-month OS of patients with non-NF1 HGGs was 81% compared to 0% in NF1 patients.

**Conclusions:** Cerebral HGGs arising in midline structures rarely occur in pediatric patients with NF1 and present with extremely poor prognosis, worse than HGGs developing in non-NF1 patients, independent of the presence or absence of H3 K27M mutation. Imaging features of aggressive biological activity on advanced MRI or amino-acid PET imaging suggest prompt neuropathological and molecular investigations.

## Introduction

Neurofibromatosis type 1 (NF1) is the most frequent hereditary cancer predisposition syndrome and is caused by germline mutations in the NF1 gene encoding for neurofibromin, a very large cytoplasmic protein that functions as a negative regulator of RAS oncoproteins (1, 2). Aberrant regulation of RAS is believed to contribute to increased cell proliferation and tumorigenesis. Indeed, patients with NF1 have an increased likelihood to develop benign and malignant tumors of the central and peripheral nervous system (3–5). The most common central nervous system (CNS) tumor in NF1 is optic pathway glioma, which occurs in ~15–20% of pediatric patients (4, 6). These tumors are often asymptomatic, very slowly progressive, and only rarely require specific treatment (4, 5), with possible spontaneous regression (7). Rarely, pediatric patients with NF1 may develop more aggressive CNS tumors, including high-grade gliomas (HGGs) (8). Of note, the overall survival (OS) of NF1 patients with HGGs has been reported to be higher than their sporadic counterparts (9).

The revised 2016 World Health Organization (WHO) classification of tumors of the CNS introduced the diffuse midline glioma (DMG) H3 K27M-mutant as a completely new entity. DMG H3 K27M-mutant arises in all midline CNS structures with the most common locations being the brainstem, thalamus, and spinal cord. Among infiltrative brainstem gliomas, the vast majority is represented by diffuse intrinsic pontine gliomas (DIPGs), in which H3 K27M mutation occurs in about 85% of cases (10). The detection of H3 K27M mutation in infiltrating midline gliomas determines an assignment to WHO grade IV (11).

The aim of this retrospective single-center study was to compare the clinical behavior of DMGs H3 K27M-mutant and non-histone mutant midline HGGs in NF1 vs. non-syndromic children and to report imaging features of NF1 HGGs.

## Materials and Methods

After approval from the Institutional Review Board (Regional Ethics committee of Liguria, Genoa, Italy), we performed a retrospective review of the electronic database of our Neuro-oncology Unit to identify potential patients admitted between 2010 and 2018. This time interval was selected because all patients diagnosed since 2010 with diffusely infiltrating astrocytic tumors arising in midline brain structures, with available pathologic tissue, underwent retrospective molecular analysis and categorization according to the revised 2016 WHO classification. In our search, we used the key terms “NF1,” “neurofibromatosis type 1,” “anaplastic astrocytoma,” “glioblastoma,” and “DMG.” Patients were included for analysis only if they (i) met neuroimaging criteria for cerebral midline location (thalamic, brainstem, or diencephalic–mesencephalic junction); (ii) underwent molecular analysis for H3 K27M; (iii) met the clinical criteria for NF1, established by the National Institutes of Health; and (iv) had genetically confirmed NF1 diagnosis. An additional search was performed to identify non-NF1 patients with cerebral DMGs as defined by WHO classification, non-histone mutant midline glioblastomas, or midline anaplastic astrocytomas who received definitive treatment at our institution during the same time period.

Patient age, diagnosis, clinical course, treatment plan, and follow-up were reviewed. In particular, progression-free survival (PFS) and OS (defined as the interval between initial diagnosis and the onset of disease progression and of death from any cause, respectively) were obtained.

Differences in PFS and OS between non-NF1 patients with and without H3 K27M mutation were evaluated by the Kaplan–Meier method and compared across groups by the log-rank test. Statistical analysis was performed by using SPSS Statistics for Mac, version 21.0 (IBM, Armonk, NY). A *p-* value of 0.05 was used to define nominal statistical significance.

## Results

Two pediatric patients were identified who met criteria for NF1 and had a DMG H3 K27M-mutant (ponto-mesencephalic gliobastoma) and a thalamic anaplastic astrocytoma, H3 K27M-wild type. During the same time period, 16 non-NF1 patients with HGGs (11 DMGs H3 K27M-mutant and 5 non-histone mutant HGGs) were treated at our institution. All subjects with DMGs H3 K27M-mutant presented mutations in the histone variant H3.3 (H3F3A). Ten non-NF1 children had been previously included in a retrospective study aimed to evaluate the diagnostic ability of 18F-dihydroxyphenylalanine (DOPA) PET and advanced MRI techniques in discriminating DMGs H3 K27M-mutant from non-histone mutant midline gliomas (12). Location and neuropathological and clinical features (treatments and outcome) of NF1 and non-NF1 patients are reported in Table 1.

**Table 1:** Summary of patient characteristics, treatments, and outcome.

**Case**	**Age at diagnosis**	**Sex**	**Histological diagnosis**	**H3K27M status**	**Location**	**WHO Grade**	**Treatments**	**Outcome**	**PFS (months)**	**OS (months)**
**NON-NF1 HIGH-GRADE GLIOMAS**										
1	12	M	GB	H3K27M-m	L-Th	IV	PS/RT/VIN+NIM/TEM	PD	6	14
2	3	F	GB	H3K27M-m	R-Th/L-Th	IV	B/RT/VIN+NIM	PD and DOD	9	13
3	10	M	GB	H3K27M-m	Pons	IV	B/RT/VIN+NIM/TEM+ETO	PD and DOD	4	6
4	12	F	GB	H3K27M-m	Medulla	IV	B/ RT/VIN+NIM/DAB+TRA	PD and DOD	5	9
5	6	F	GB	H3K27M-m	R-DMJ	IV	B/RT/TEM+BEV	PD and DOD	4	8
6	6	M	GB	H3K27M-m	Pons	IV	B/RT/VIN+NIM	PD	6	10
7	7	M	AA	H3K27M-m	Pons	IV	B/RT/VIN+NIM/IRI/SIR	PD and DOD	7	10
8	8	F	AA	H3K27M-m	Pons	IV	B/RT/VIN+NIM	PD and DOD	6	12
9	16	F	AA	H3K27M-m	R-DMJ	IV	B/RT/TEM+BEV	PD and DOD	5	6
10	10	F	AA	H3K27M-m	R-Th	IV	B/RT/TEM+BEV	PD and DOD	10	18
11	3	F	AA	H3K27M-m	R-Th	IV	B/RT/TEM	PD and DOD	4	8
12	6	M	GB	H3K27M-wt	L-Th	IV	B/RT/TEM+BEV	PD and DOD	6	10
13	9	M	GB	H3K27M-wt	L-Th	IV	PS/RT/CAR+VC/TEM/BEV+ETO	PD and DOD	4	7
14	9	M	GB	H3K27M-wt	R-Th	IV	B/RT/TEM+BEV/POM/ETO	PD and DOD	5	11
15	11	M	AA	H3K27M-wt	R-Th/L-Th	III	B/RT/TEM	PD and DOD	6	12
16	17	F	AA	H3K27M-wt	R-Th/L-Th	III	PS/RT/TEM+BEV/VIN+RAP	PD and DOD	8	12
**NF1 HIGH-GRADE GLIOMAS**										
1	11	F	GB	H3K27M-m	L-Pons midbrain	IV	B/RT/VIN+NIM/ETO+TEM	PD and DOD	3	7
2	13	F	AA	H3K27M-wt	R-Th/L-Th	III	B/RT/TEM+VIN	PD and DOD	3	5

*PFS, progression-free survival; OS, overall survival; M, male; F, female; GB, glioblastoma; AA, anaplastic astrocytoma; m, mutant; wt, wild type; R, right; L, left; Th, thalamus; DMJ, diencephalic-mesencephalic junction; B, biopsy; PS, partial surgery; RT, radiotherapy; VIN, vinorelbine; NIM, nimotuzumab; TEM, temozolomide; ETO, etoposide; DAB, dabrafenib; TRA, trametinib; BEV, bevacizumab; IRI, irinotecan; SIR, sirolimus; CAR, carboplatin; VC, vincristine; POM, pomalidomide; RAP, rapamycin; PD, progressive disease; DOD, death of disease*.

The two pediatric patients with NF1 presented a PFS of 3 months and an OS of 5 and 7 months. Median PFS and OS in non-NF1 children were, respectively 6 and 10 months (PFS range 4–10 months, OS range 6–18 months). In detail, median PFS and OS were 6 and 10 months in non-NF1 DMGs H3 K27M-mutant (PFS range, 4–10 months; OS range, 6–18 months), and 6 and 11 months in non-histone mutant midline HGGs (PFS range, 4–9 months; OS range, 7–14 months).

Leptomeningeal dissemination was diagnosed during follow-up in one NF1 DMG H3 K27M-mutant. Among non-NF1 HGGs, it was revealed in 3 out of 11 patients with DMGs H3 K27M-mutant and in 1 out of 5 non-histone mutant midline HGGs. In all subjects, leptomeningeal dissemination was better recognizable and much more prominent in the spinal region; none of the patients presented leptomeningeal dissemination at admission.

No statistically significant differences in terms of PFS and OS emerged between non-NF1 subjects with DMGs H3 K27M-mutant and non-histone mutant midline HGGs [χ^2^(2) = 0.114, *p* < 0.736 and χ^2^(2) = 0.000003, *p* < 0.989, respectively]. While the small number of patients with NF1 precludes formal statistical analysis, 75% of subjects with non-NF1 HGGs presented a PFS >4 months compared to 0% in NF1 patients. The 8-month OS of patients with non-NF1 HGGs was 81% compared to 0% in NF1 patients. A description of each of the two NF1 cases follows.

### Case 1

This 11-year-old female presented a few days' history of headache, vomiting, difficult writing, dysphagia, dysarthria, and right-sided hemiparesis. NF1 had already been diagnosed on a clinical basis and through the identification of the de novo c.6792C>A variant in the neurofibromin gene, determining the substitution of a tyrosine with a stop codon (p.Tyr2264*) resulting in a protein lacking 34 amino-acids (13).

**Figure 1:**
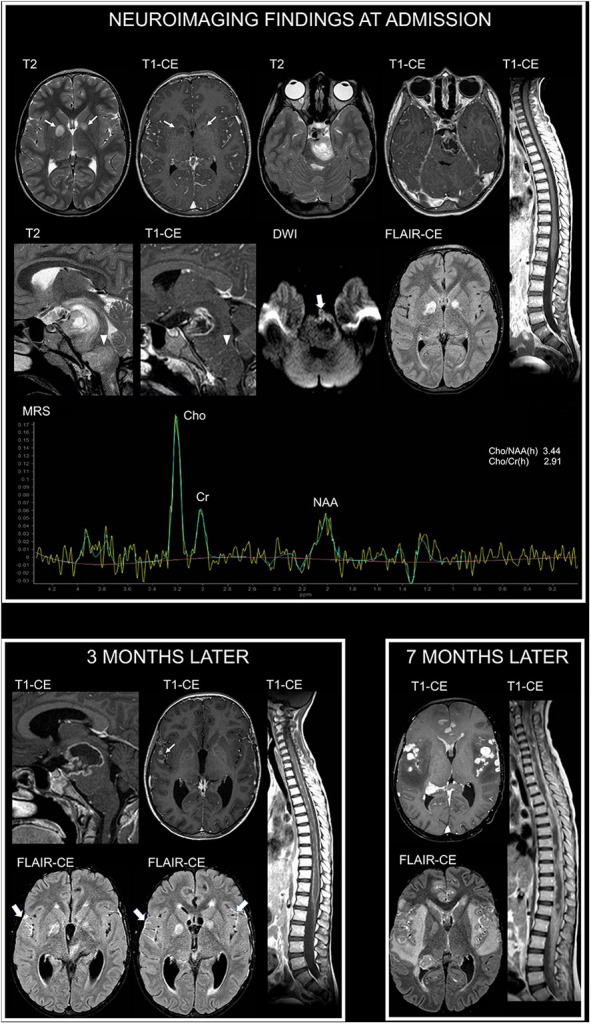
Neuroimaging findings in NF1 patient 1. At admission, brain axial T2-weighted and contrast-enhanced (CE) T1-weighted images show focal areas of signal abnormalities without contrast enhancement in the globus pallidus bilaterally, in keeping with typical unidentified bright objects (thin arrows). Additional brain axial and sagittal T2-weighted and CE T1-weighted images show mass lesions with a central necrotic area and irregular rim enhancement in the left ponto-mesencephalic region, along with (arrowheads) an adjacent expansile lesion involving the medulla without contrast enhancement. Diffusion-weighted imaging (DWI) shows reduced diffusivity along the ventrolateral margin of the ponto-mesencephalic lesion (thick arrow). Post-contrast fluid attenuated inversion recovery (FLAIR-CE) image does not reveal cerebral leptomeningeal dissemination. Sagittal CE T1-weighted image of the spine does not show secondary lesions. Single-voxel magnetic resonance spectroscopy (MRS) with an echo time of 144 ms of the ponto-mesencephalic lesion shows prominent increase of Cho/NAA and Cho/Cr ratios. Three months later, following radiotherapy and first-line chemotherapy treatment with vinorelbine and nimotuzumab, sagittal brain CE T1-weighted image shows increased extension of the necrotic component within the ponto-mesencephalic lesion. Axial CE T1-weighted and post-contrast FLAIR images (FLAIR-CE) demonstrate leptomeningeal contrast enhancement, in keeping with secondary disseminati5on, along the sylvian fissures (thin arrow and thick arrows). Leptomeningeal dissemination is more evident on sagittal CE T1-weighted image of the spine. Seven months after diagnosis, following second-line treatment with etoposide and temozolomide, axial CE T1-weighted and FLAIR-CE images show marked increase of nodular leptomeningeal dissemination with extensive brain edema and subependymal dissemination. Sagittal CE T1-weighted image of the spine show massive secondary involvement around and within the spinal cord.

MRI at admission showed (in addition to unidentified bright objects located in the deep cerebellar white matter and basal ganglia) a mass lesion with an irregular central necrotic area in the left ponto-mesencephalic region (Figure 1). Diffusion-weighted imaging (DWI) showed reduced diffusivity along the ventrolateral margin of the lesion (minimum absolute ADC value: 0.69 × 10^−3^ mm^2^/s). Magnetic resonance spectroscopy (MRS), performed using a single-voxel point resolved spectroscopy technique with an echo time of 144 ms, a repetition time of 2000 ms, and 128 signal averages, showed a Cho/NAA peak-height ratio of 3.44 and Cho/Cr ratio of 2.91 (Figure 1). An additional adjacent expansile lesion without contrast enhancement or necrotic areas was found in the medulla. The patient underwent biopsy of the ponto-mesencephalic lesion and neuropathology demonstrated a diffuse midline glioma H3K27M-mutant (glioblastoma). She was started with focal radiotherapy in association with medical treatment with vinorelbine and nimotuzumab.

Follow-up MRI performed 3 months later, following first-line treatment, revealed brain and spine leptomeningeal dissemination, not present at diagnosis, in keeping with progressive disease (Figure 1). The primary lesion demonstrated an increased necrotic component with perilesional edema, suggestive of radiation induced changes. Clinically, the patient presented global deterioration of the neurological status, and she underwent a cerebrospinal fluid diversion due to symptomatic hydrocephalus. Subsequently, she was started with a second-line chemotherapy course with etoposide and temozolomide.

She was re-evaluated after the first two cycles (5 months since diagnosis) with brain and spinal imaging, demonstrating further increase of the secondary dissemination; the primary ponto-mesencephalic lesion presented decreased volume of the necrotic component, supporting the diagnosis of radiation-induced changes, and no evidence of local progression.

Clinical conditions worsened and a subsequent brain MRI performed 7 months since diagnosis demonstrated a massive brain and spine leptomeningeal dissemination with diffuse brain edema (Figure 1). The patient died a few days later.

### Case 2

**Figure 2:**
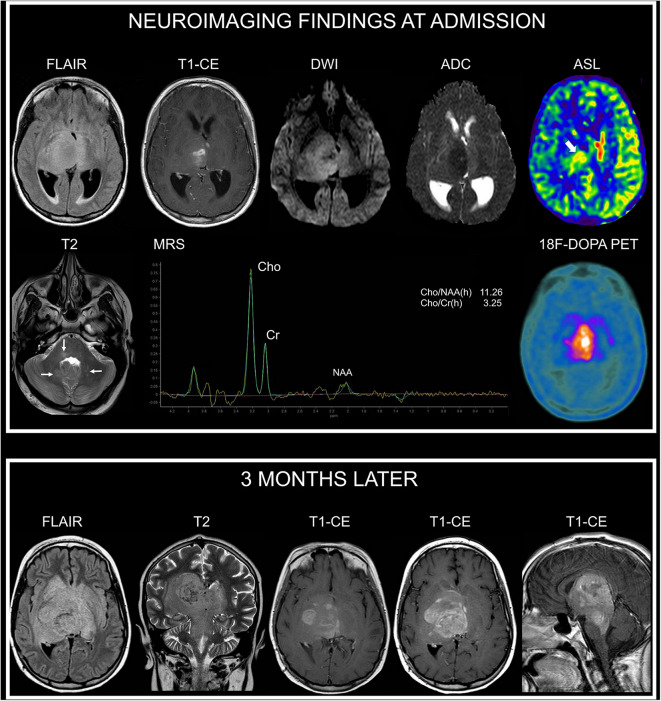
Neuroimaging findings in NF1 patient 2. At admission, brain axial FLAIR and CE T1-weighted images show an infiltrating and expansile lesion with epicenter in the right thalamus, partially involving the contralateral thalamus, with irregular contrast enhancement. DWI and corresponding apparent diffusion coefficient (ADC) map show restricted diffusivity of the right thalamic portion of the lesion. Arterial spin labeling (ASL) perfusion-weighted imaging clearly demonstrates increased perfusion of the lesion (thick arrow). Axial T2-weighted image shows small focal hyperintense areas located in the dorsal pons and deep cerebellar white matter (thin arrows) in keeping with typical unidentified bright objects. Single-voxel MRS with an echo time of 144 ms of the right thalamic region shows marked increase of Cho/NAA and of Cho/Cr ratios. 18F-DOPA PET clearly demonstrates markedly increased uptake of the lesion. Three months later, following radiotherapy and first-line chemotherapy treatment with temozolomide and vinorelbine, axial FLAIR and coronal T2-weighted images show increased extension of the infiltrating components in the deep cerebral regions with prominent involvement of the brainstem (midbrain and dorsal pons). Axial and sagittal CE T1-weighted images show concomitant marked increase of contrast enhancement. There was no evidence of leptomeningeal dissemination.

A 13-year-old female presented with a recent history of headache, episodes of vomiting, and left-sided hemiparesis. Clinical examination revealed the presence of multiple café-au-lait macules and axillary and inguinal freckling. She was found to carry the c.5705C>A variant, which determines an amino-acid substitution threonine with a lysine (p.Thr1902Lys). This variant was transmitted by her affected mother. Brain and spine MRI at admission showed (in addition to unidentified bright objects in the deep cerebellar white matter, dorsal pons, and in the globus pallidus bilaterally) an expansile and infiltrating lesion with epicenter in the right thalamus extending to the contralateral thalamus characterized by irregular contrast enhancement (Figure 2).

On DWI, the minimum absolute ADC value of the lesion was 0.68 × 10^−3^ mm^2^/s; MRS demonstrated a Cho/NAA peak-height ratio of 11.26 and Cho/Cr ratio of 2.29. Perfusion-weighted imaging, performed with pulsed arterial spin labeling (ASL) technique, demonstrated a relative tumoral maximum cerebral blood flow (rCBF max) of 1.5 (obtained by normalizing the tumoral CBF max by a blood flow measurement from the normal appearing contralateral gray matter in the temporal lobe) (Figure 2). There was no evidence of leptomeningeal dissemination. The patient also underwent cerebral 18F-DOPA PET imaging demonstrating markedly increased uptake of the lesion, with a maximum tumor/striatum ratio of 1.35 (Figure 2). She underwent a stereotaxic biopsy and neuropathology demonstrated an anaplastic astrocytoma, H3K27M-wild type. She underwent treatment with radiotherapy and chemotherapy with temozolomide and vinblastine.

Follow-up MRI performed 3 months later following first-line treatment documented a significant disease progression without leptomeningeal dissemination (Figure 2). Her neurological status deteriorated rapidly and the patient died 5 months after diagnosis.

## Discussion

Pediatric HGGs are a relatively rare group of CNS neoplasms with an aggressive behavior and poor prognosis (14, 15). About 50% of pediatric cerebral HGGs arise in midline structures such as the brainstem and in particular the pons, the thalamus, and rarely the cerebellum (14). In the revised 2016 WHO classification of tumors of the CNS, and in the recent guidelines of the cIMPACT-NOW (the Consortium to Inform Molecular and Practical Approaches to CNS Tumor Taxonomy) Working Committee 3, the DMG H3 K27M-mutant has been recognized as a new diagnostic entity that should only include infiltrating gliomas arising in midline structures (11, 16).

H3 K27M mutation results in substitution of the amino-acid lysine to methionine at residue 27, inducing unique gain-of-function mechanisms that lead to global reduction of H3 with trimethylated lysine 27 (H3K27me3). Even though the precise role of H3 K27M mutation in tumorigenesis remains not clearly defined, functional analysis has underscored its contribution to inhibition of autophagy and abnormal cell-cycle control (17–20). A recent study revealed also that H3 K27M mutation accelerates brainstem tumorigenesis of HGGs from neonatal progenitor cells (21). Genomic analysis of DMGs H3 K27M-mutant has demonstrated a number of cooperating genetic alterations. In particular, these tumors frequently present TP53 and ATRX mutations (14, 20). In addition, the most recurrently mutated gene in DIPGs H3 K27M-mutant after the histone variants is the ACVR1, which encodes the activin A receptor type-1 transmembrane protein (20, 22).

In the setting of NF1, pediatric HGGs have been described (4, 9, 14, 23–25) with a prevalence ranging from 0.28 to 5% (23). NF1-related pediatric HGGs share genetic alterations of TP53 and CDKN2A with non-NF1 patients (26). A recent study (24) also demonstrated that NF1 HGGs “harbor frequent mutations of ATRX associated with Alternative Lengthening of Telomere, and are enriched in genetic alterations of transcription/chromatin regulation and PI3 kinase pathways.” Frequent mutations of ATRX drive aggressiveness in NF1 gliomas. Furthermore, “loss of ATRX in NF1 HGGs is unique when considered within the genetic contexts associated with ATRX mutations in sporadic gliomas,” in which they are typically associated with pediatric H3 K27M-mutant DMGs (24).

Of note, due to their rarity, a child with a clinical diagnosis of NF1 and HGG should be investigated for constitutional mismatch-repair deficiency (CMMRD) if an NF1 mutation has not been previously identified (27). CMMRD frequently displays features reminiscent of NF1 (28). Genetic confirmation of NF1 is therefore mandatory for genetic counseling to families and because alternative therapies are available for CMMRD-associated HGGs (27, 28). In particular, these patients may benefit from immunotherapy with antibodies against the programmed cell death protein-1, whereas temozolomide should be avoided (27, 29). In our patients, genetic analysis of NF1 was performed, identifying in patient 1 a c.6792C>A variant in the NF1 gene determining a p.Tyr2264^*^ premature termination with skipping of exon 37 resulting in a protein lacking 34 amino acids (13), while patient 2 was carrying the c.5705C>A variant that determines an amino-acid substitution p.Thr1902Lys. This variant is not present in gnomAD (Genome Aggregation Database, https://gnomad.broadinstitute.org/); it involves a highly conserved amino acid and was transmitted by her affected mother. Eight different prediction tools, which predict the possible impact of an amino-acid substitution on the structure and function of a human protein using straightforward physical and comparative considerations, indicate that the variant has deleterious (D) effect (Appendix).

It is well-known that neurofibromin has an important function in cancer development and progression. Bi-allelic loss of neurofibromin confers a growth advantage in astrocytes *in vitro* and is required for tumor formation (30, 31).

HGGs involving midline structures in the setting of NF1 are extremely rare, with few pediatric patients reported so far (4, 9, 23, 25). In these prior reports, the association of midline HGGs with NF1 was mainly based on a clinical NF1 diagnosis, thus potentially not excluding a CMMRD.

Of note, one of our NF1 patients presented a DMG H3 K27M-mutant and currently represents the first description in the literature where this type of tumor is reported in association with a genetically confirmed diagnosis of NF1. The rarity of DMGs H3 K27M-mutant in NF1 patients is underlined by a recent study where genomic profile of 59 gliomas (22 children, 33 adults) was evaluated. Remarkably, H3.3 histone variants were absent in all 59 cases (25).

Regarding our NF1 patients, none of them presented an optic pathway glioma or a mass lesion in another district, or received prior radiotherapy; typical unidentified bright objects were present. In both cases, neoplasms demonstrated an unexpected aggressive behavior, with no response to conventional therapies and rapid leptomeningeal dissemination in one subject, with a PFS of 3 months and an OS of 7 and 5 months.

Future studies are required in order to explore alternative therapeutic approaches, including immunotherapy (32), in NF1 patients with DMGs.

Neuroimaging studies can play a pivotal role in suggesting HGG, thus recommending biopsy sample in NF1 subjects. Both patients presented areas of restricted diffusivity within the lesions. MRS demonstrated pathologic increase of Cho/NAA and Cho/Cr ratios. One NF1 subject underwent both MRI perfusion imaging with ASL and molecular imaging with 18F-DOPA PET, demonstrating increased perfusion and markedly increased amino-acid uptake. All these techniques have been demonstrated to add significant information in discriminating low-grade from high-grade cerebral gliomas, both midline and off-midline, providing non-invasive microstructural, microvascular, and metabolic information (12, 33, 34). In detail, DWI enables estimation of brain tumor cellularity, and in our NF1 subjects, reduced diffusivity of the lesions was suggestive of increased cell density.

ASL allows quantification of cerebral blood flow correlated with microvascular density, providing additional non-invasive information of pediatric brain gliomas aggressiveness, as demonstrated in case 2. MRS allows the estimation of normal and abnormal brain metabolites, indicating loss of neuronal integrity and increased turnover of myelin (33). Both subjects presented an MRS pattern in keeping with increased biological activity of the lesions. Among amino-acid PET tracers, 18F-DOPA has demonstrated a high degree of correlation with tumor grade in pediatric infiltrative astrocytomas (12, 34). Increased 18F-DOPA uptake is related to an overexpression of the L-type amino-acid transporter 1, within highly proliferative tumoral components (34, 35). Advanced MR imaging studies and/or molecular amino-acid PET imaging are therefore recommended in those NF1 subjects with suspected aggressive lesions on conventional MRI in order to provide additional information and increase diagnostic confidence. Of note, prior studies have reported that thalamic localization, symptoms at diagnosis, and diffusion restriction on MRI are elements suggestive of a high-grade tumor in NF1 subjects (23, 25), as confirmed in our study.

In the non-NF1 population, we did not find statistically significant differences in terms of PFS and OS between DMGs H3 K27-mutant and non-histone mutant midline HGGs. In the revised 2016 WHO classification of tumors of the CNS, “for DMGs in general, the finding of an H3 K27M mutation, confers a worse prognosis than that of wildtype cases” (11). However, as reported by recent researches, non-unequivocal findings are emerging regarding the prognostic role of H3 K27M mutation in DMGs, in accordance with our results. For instance, recent studies highlighted that H3 K27M-wild-type DIPGs (~15% of the biopsied population) shared the same unfavorable prognosis as H3 K27M-mutant DIPGs (36, 37), independently of their underlying histological tumor grading. Survival comparison between H3 K27M-mutant and wild-type midline gliomas in adults also demonstrated that survival may be similar or possibly improved if the mutation is present (38, 39). Further and larger prospective studies are therefore recommended to better define the prognostic significance of H3 K27M mutation in DMGs, as also suggested in a recent meta-analysis (40).

In both NF1 and non-NF1 HGGs, leptomeningeal dissemination during treatment response evaluation was revealed in five subjects, both H3K27M-mutant and wild type, with no such evidence at admission. This finding was clearly evaluable and more prominent in the spinal compartment, when compared to the brain. Of note, in the NF1 patient, the appearance of leptomeningeal dissemination did not show a concomitant primary lesion progression. Overall, these findings advise whole brain and spine MRI studies in DMGs, at admission and during follow-up, for a complete evaluation of the disease status.

In the setting of NF1, leptomeningeal dissemination of pediatric gliomas is an extremely rare event. We found only one description of a pediatric patient with an HGG and diagnostic criteria of NF1, where evidence of tumor dissemination was reported (23). An additional single patient with NF1 and a midline low-grade glioma (a pilocytic astrocytoma) with secondary dissemination to the brain has been previously described (41).

Among the limitations of our study, we are aware of its retrospective nature and of the relatively small sample of patients; however, we included only pediatric patients with HGGs arising in midline brain structures, molecularly classified, and NF1 subjects with genetic diagnosis, which are extremely rare, particularly for a single center. The limited number of NF1 patients did not allow performing formal statistical analysis, and further multicenter studies with larger samples of patients are needed to extend knowledge in this field. Additional expression studies on H3 K27M-mutant DMGs in NF1 patients are also needed to evaluate up- or down-regulated genes and pathways that might represent a potential therapeutic target.

In conclusion, according to our experience, HGGs arising in midline brain structures in NF1 pediatric patients present an aggressive behavior and an extremely poor prognosis, worse than HGGs in non-NF1 patients, independent of the presence or absence of H3 K27M mutation. Lesions in evocative regions and with features of increased biological activity on advanced MRI or molecular amino-acid PET imaging may alert clinicians, suggesting prompt neuropathological and molecular investigations.

## Data Availability Statement

The raw data supporting the conclusions of this article will be made available by the authors, without undue reservation, to any qualified researcher.

## Ethics Statement

The studies involving human participants were reviewed and approved by the Regional Ethics committee of Liguria, Genoa, Italy. Written informed consent to participate in this study was provided by the participants' legal guardian/next of kin. Written informed consent was obtained from the minors' legal guardian/next of kin for the publication of any potentially identifiable images or data included in this article.

## Author Contributions

FG and CM: conceptualization, data curation, and writing—original draft. FM: conceptualization, investigation, and writing—review and editing. AV and GP: conceptualization and data curation. AP: conceptualization, formal analysis, and investigation. DT: conceptualization, formal analysis, and methodology. MD: conceptualization, data curation, and resources. VC: conceptualization, formal analysis, and writing—original draft. MG, AR, and GM: conceptualization, data curation, supervision, and writing—review and editing. All authors contributed to manuscript revision, and read and approved the submitted version.

## Conflict of Interest

The authors declare that the research was conducted in the absence of any commercial or financial relationships that could be construed as a potential conflict of interest.

## Appendix

SIFT, Sorting Intolerant from Tolerant (https://sift.bii.a-star.edu.sg/): D; PolyPhen-2, Polymorphism Phenotyping v2 (http://genetics.bwh.harvard.edu/pph2/): HumDiv = 1.000 - HumVar = 0.999 (probably damaging); CADD, Combined Annotation Dependent Depletion (https://cadd.gs.washington.edu/snv): 34; DANN, omicX, deep neural network (https://omictools.com/dann-tool): 0.992; FATHMM, Functional Analysis through Hidden Markov Models (v2.3) (http://fathmm.biocompute.org.uk/): −4.23; GERP++, omicX (https://omictools.com/gerp-tool): 5.3; Mutation Taster (http://www.mutationtaster.org/): D, D; PROVEAN, Protein Variation Effect Analyzer (http://provean.jcvi.org/:D).

Furthermore this variant is not present in ClinVar; the C>G change (c.5705C>G; p.Thr1902Arg - https://www.ncbi.nlm.nih.gov/clinvar/variation/404539) and the C>T change (c.5705C>T; p.Thr1902Met - https://www.ncbi.nlm.nih.gov/clinvar/variation/187560) in the same position (rs786203824; chr17: 29657472; GRCh37.p13) have been defined in ClinVar of uncertain significance.
